# Plastic responses of survival and fertility following heat stress in pupal and adult *Drosophila virilis*

**DOI:** 10.1002/ece3.8418

**Published:** 2021-12-01

**Authors:** Benjamin S. Walsh, Steven R. Parratt, Natasha L. M. Mannion, Rhonda R. Snook, Amanda Bretman, Tom A. R. Price

**Affiliations:** ^1^ Institute of Integrative Biology University of Liverpool Liverpool UK; ^2^ Department of Zoology Stockholm University Stockholm Sweden; ^3^ School of Biology Faculty of Biological Sciences University of Leeds Leeds UK

**Keywords:** climate change, plasticity, reproduction, sterility

## Abstract

The impact of rising global temperatures on survival and reproduction is putting many species at risk of extinction. In particular, it has recently been shown that thermal effects on reproduction, especially limits to male fertility, can underpin species distributions in insects. However, the physiological factors influencing fertility at high temperatures are poorly understood. Key factors that affect somatic thermal tolerance such as hardening, the ability to phenotypically increase thermal tolerance after a mild heat shock, and the differential impact of temperature on different life stages are largely unexplored for thermal fertility tolerance. Here, we examine the impact of high temperatures on male fertility in the cosmopolitan fruit fly *Drosophila virilis*. We first determined whether temperature stress at either the pupal or adult life history stage impacts fertility. We then tested the capacity for heat‐hardening to mitigate heat‐induced sterility. We found that thermal stress reduces fertility in different ways in pupae and adults. Pupal heat stress delays sexual maturity, whereas males heated as adults can reproduce initially following heat stress, but become sterile within seven days. We also found evidence that while heat‐hardening in *D*. *virilis* can improve high temperature survival, there is no significant protective impact of this same hardening treatment on fertility. These results suggest that males may be unable to prevent the costs of high temperature stress on fertility through heat‐hardening, which limits a species’ ability to quickly and effectively reduce fertility loss in the face of short‐term high temperature events.

## Graphical abstract

Increasing heat waves as a result of climate change will kill and sterilize individuals. We need to know whether a short‐term sublethal temperature stress, known as “heat‐hardening,” prior to heat stress can allow individuals to plastically prevent these costs. We find that heat‐hardening improves survival but not fertility at high temperatures, suggesting that reproduction is not as flexible to heat waves as survival.

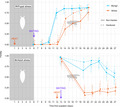

## 1 INTRODUCTION

Climate change is increasing the frequency of extreme temperature events (Christidis et al., 2015). A major research priority is to assess which organisms will be able to maintain fitness and cope with the changing climate. Initial efforts to explore the impact of rising temperatures on biodiversity mostly considered how thermal stress affects survival (Deutsch et al., 2008; Kellermann et al., 2012; Pinsky et al., 2019). While the impact of climate change on survival is clearly important, it has also been known for around a century that fertility is vulnerable to high temperatures in some species (Cowles, 1945; Young & Plough, 1926). In this paper, we use fertility to mean the ability to produce offspring, the direct opposite of sterility. We use this definition because complete sterility has the potential to be extremely important in a warming world (Parratt et al., 2021; Walsh et al., 2019; van Heerwaarden & Sgrò, 2021). Heat‐induced sterility occurs across diverse taxa including crops (Matsui et al., 1997) and livestock (Karaca et al., 2002), so species where fertility is lost at temperatures far below the lethal limit may represent both a major economic and conservation concern (Walsh et al., 2019) with potentially worrying implications for humanity's resilience against climate change. Fertility loss is generally sex‐specific, with males often more sensitive to fertility loss than females (Iossa, 2019; Sales et al., 2018; Walsh et al., 2021; Zwoinska et al., 2020). Recent work has found that the highest temperatures *Drosophila* species are found at worldwide is strongly correlated to laboratory measurements of their lethal temperature, or the temperature at which males lose fertility, whichever is the lower (Parratt et al., 2021; van Heerwaarden & Sgrò, 2021). This suggests that species distributions may often be restricted by their upper thermal limits to fertility in nature. However, we still know relatively little about the physiological factors that affect fertility loss at high temperatures.

In holometabolous insects, it is widely known that survival at high temperatures can be affected by the life stage at which thermal stress occurs (Moghadam et al., 2019; Zhang et al., 2015). Studies on heat‐induced sterility in males typically use either a single long‐term stress across age‐groups (Porcelli et al., 2016; Rohmer et al., 2004), or an acute stress to individuals from a single age‐group (Jørgensen et al., 2006, 2021; Sales et al., 2018; Walsh et al., 2021). However, it has recently been shown in the flour beetle *Tribolium castaneum* that the extent of male fertility loss depends on the life stage exposed to thermal stress (Sales et al., 2021). Here, pupal and immature adults show the highest sterility after thermal stress as compared with larval and mature adults. This study reveals a critical period in the life cycle of *T*. *castaneum* where fertility is particularly vulnerable to heat stress of immature individuals. In order to uncover any general patterns in thermal sensitivity of fertility across life stages, research should examine this across species.

One way organisms can cope with thermal stress is to plastically invest resources into thermal protection after receiving a signal that the risk of extreme high temperatures has increased. For example, exposure to a short‐term moderately stressful sublethal heat can cause organisms to make physiological changes that allow them to better survive extreme temperatures (Loeschcke & Hoffmann, 2007; Moghadam et al., 2019). This response is called heat‐hardening and is widespread in animals and plants (Bilyk et al., 2012; Moghadam et al., 2019; Neuner & Buchner, 2012). The positive impacts of hardening in ectotherms are generally thought to occur through the upregulation of heat shock proteins such as HSP70 (Sørensen et al., 2001). When the individual thereafter experiences extreme temperatures, the increased concentration of heat shock proteins reduces the thermal damage. Hardening has been shown to mitigate the deleterious effects of high temperatures on a multitude of traits, including survival (Heerwaarden et al., 2016; Moghadam et al., 2019) and the ability to locate resources such as food or mating sites (Loeschcke & Hoffmann, 2007). In the fruit fly *Drosophila melanogaster*, individual survival is improved at high temperatures through hardening; however, the amount of protection provided changes depending on the life stage measured (Moghadam et al., 2019). In this case, pupae show strong protection through heat‐hardening, whereas adults’ hardening capacity is minimal. Clearly, a full understanding of heat‐hardening itself is difficult without examining multiple life stages.

While the capacity of individuals to improve survival through heat‐hardening is widespread, it remains unclear whether individuals can utilize hardening to mitigate heat‐induced sterility. Some studies suggest that there is a trade‐off between hardening and reproduction (Krebs & Loeschcke, 1994), but other examples found hardening improves mating behavior (Sambucetti & Norry, 2015) and, in a few species, heat‐hardened individuals show greater offspring production after thermal stress (Jørgensen et al., 2006; Sarup et al., 2004). Heat‐induced sterility occurs at sublethal temperatures in many organisms (Walsh et al., 2019), including ~44% of a panel of 43 *Drosophila* species (Parratt et al., 2021). So it is likely that, in the marginal populations of particularly vulnerable species, a male's fitness could be greatly improved by maintaining fertility at sublethal stress temperatures. If males can plastically harden to prevent fertility loss at extreme temperatures, then populations may have the capacity to better cope with sublethal but stressful heat events.

Here, we explore the impact of high temperatures on male fertility in the cosmopolitan fruit fly *Drosophila virilis*, an extremely widespread model species. Critically, it has previously been demonstrated that male *D*. *virilis* can be sterilized by thermal stress well below their lethal temperature limit (80% of adult males sterile after four hours at 35°C, 80% of adult males dead after four hours at 38°C) (Parratt et al., 2021; Walsh et al., 2021). This sterilization of males at survivable temperatures makes *D*. *virilis* an ideal species to look for heat‐hardening of fertility. We test the impact of temperature stress on fertility across life‐history stages, heating individuals as either pupae or adults. Further, we demonstrate the capacity for heat‐hardening to improve survival at extreme temperatures and subsequently test if this hardening response can also mitigate heat‐induced sterility. Importantly, we measure how fertility changes over an individual's age, to better understand the long‐term fitness implications of thermal stress and hardening at different life stages.

## 2 MATERIALS AND METHODS

In overview, we test if heat shocks experienced during pupal and adult life‐history stages result in male sterility. We also test if a brief period of heat‐hardening can ameliorate these effects. In a series of experiments, adult and pupal male *D*. *virilis* were exposed to a 1‐h heat‐hardening treatment followed immediately by a 4‐hour heat stress. They were then immediately assayed for survival, and their fertility was subsequently measured over 1–2 weeks to reveal temporal patterns in fertility loss and restoration. We chose a 4‐hour stress because midday rises to high temperature are relatively common (Geletič et al., 2020), and we think it is ecologically reasonable that a fly in nature might be exposed to these conditions for a few hours. Moreover, it is an experimentally tractable time period, and previous work has demonstrated this method can create male sterility in many *Drosophila* species, including *D*. *virilis* (Parratt et al., 2021; Walsh et al., 2021).

### 2.1 Animal stock maintenance

Stocks of *Drosophila virilis* (Cambridge Fly Facility StrainvS‐4, isolated in 1991) were kept in a temperature‐controlled room at 23°C, 12:12 L:D, and ambient humidity. Although a long‐term laboratory stock, this stock was included in a recent analysis of upper thermal limits from 36 *Drosophila* species that found no significant association between time in culture and any upper thermal limit (Parratt et al., 2021), suggesting it is a reasonable model for the species. Stocks were maintained at moderate density (50–100 flies per 300‐ml bottle culture, representing a low level of larval crowding) on “Propionic” medium (10 g agar, 20 g yeast extract, 70 g cornmeal, 10 g soya flour, 80 g malt extract, 22 g molasses, 14 ml 10% nipagin, 6 ml propionic acid, 1000 ml H_2_O). Ovipositing adults were tipped to new food every week to prevent overlapping generations and were replaced with fresh sexually mature adult flies every 4–6 weeks.

### 2.2 Pupal heat stress

#### 2.2.1 Survival

Pupae were collected from stock vials within 24 h of pupation, allocated to vials of 20 pupal flies. Three vials were allocated to each treatment (giving 60 flies total per treatment, ~30 males, as sex cannot be determined in young pupae). These vials were randomly assigned to 3D‐printed floating racks into preheated water baths (Grant TXF200) for 1 h at either a control nonhardening temperature at 23°C (“no hardening”) or a range of hardening temperatures (“hardening”: 34, 35, and 36°C). These are nonlethal pupal temperatures that also do not significantly sterilize males (Walsh et al., 2021). After this hardening treatment, they were immediately moved into different preheated water baths for 4 h at either 23°C (“benign”) or at a range of five sublethal to lethal temperatures (37, 38, 39, 40, 41°C: “stress”). Immediately following treatment, vials were returned to benign conditions (23°C) and emerging individuals were collected and sexed. This allowed us to assess survival of pupae at extreme temperatures and gave us an idea of whether survival may be sex specific. However, as we were unable to determine the sex of the pupae prior to stress, we could not explicitly test for sex differences in survival thermal tolerance.

#### 2.2.2 Fertility

Pupae were allocated to 3D‐printed floating racks in preheated water baths set to 23°C (“no hardening”) or 36°C (“hardening”) for 1h as above. Immediately following hardening, they were transferred into preheated water baths at 23°C (“benign”) or 38°C (“stress”), chosen as the highest temperature not resulting in significant mortality from a prior study (Walsh et al., 2021). After four hours at their treatment temperature, vials were subsequently removed from the water baths and returned to benign temperatures (23°C). Emerging males were collected and immediately moved into individual vials with 4 sexually mature virgin female partners each. These groups were moved into new vials every 2 days for 7 times, giving a total of 8 vials across 16 days where fertility was recorded. Age at reproductive maturity (ARM) was taken as the time‐point (days postpupation) of a males’ first fertile vial. Estimates of *Drosophila* survival rates in nature suggest 16 days represents a substantial portion of their expected adult lifespan (Powell, 1997). Males were deemed as qualitatively fertile at any given time‐point if there was evidence of larvae present in the vial (either via direct observation of larvae or observing larval tracks in the food).

### 2.3 Adult heat stress

#### 2.3.1 Survival

Virgin males and females (all 7 days old) were separated and allocated to vials of 10 flies per vial of their respective sex. These vials were randomly allocated to 3D‐printed floating racks in preheated water baths for one hour at a hardening temperature at 23°C (“no hardening”) or 33°C (“hardening,” determined as the highest temperature in which no sterility is observed (Parratt et al., 2021)). After this hardening treatment, vials were immediately moved into different preheated water baths for four hours at either 23°C (“benign”) or 38°C (“stress,” determined as lowest lethal temperature from (Parratt et al., 2021)). Immediately following treatment, vials were returned to benign conditions (23°C) and left for 24 h to ensure that any flies that were immobilized by heat but not killed could recover. After 24 h, the number of surviving males and females from each treatment was assessed.

#### 2.3.2 Fertility

Virgin males were allocated to vials (10 per treatment) and treated in preheated water baths at 23°C (“nonhardening”) or 33°C (“hardening”) for 1h as above. Immediately following heat‐hardening, flies were transferred into preheated water baths at 34°C for a further 4 hours (“stress,” chosen as the lowest whole‐degree Celsius temperature at which *D*. *virilis* are sterilized (Parratt et al., 2021)). Vials were subsequently removed from the water baths and males were placed in new individual vials with 4 virgin female partners each. Previous experiments have shown that, when stressed as adults, male *D*. *virilis* initially retain fertility for several days and then become sterilized (Parratt et al., 2021). Hence, unlike our assay with pupal stress flies, we did not passage males to new vials every 2 days immediately. Instead, we gave males an initial 7‐day period in a single vial with 4 females. We then gave each male 4 new virgin females and passaged each group every 2 days for 4 times.

### 2.4 Statistical analyses

Measuring fertility, which is a long‐term adult trait when individuals are heated during different life stages, introduces significant temporal biases. We decided to measure fertility from the earliest possible time‐point poststress and continue to measure over time. This allowed us to capture any visible loss/regain of fertility. Flies do not breed as pupae, so fertility cannot be measured immediately following heat stress during this stage. Therefore, in order to understand how these responses change depending on life stage, we measured fertility over a substantial period of time after stress for both pupae and adults. Due to the inherent differences this introduced, we analyzed pupal and adult heat stress separately, so comparisons of responses between stages can only be qualitative.

Data were analyzed using variations on linear models. We assessed model fit by plotting patterns in residuals against fits and against predictors. All statistical analyses were completed in R (version 3.5.0), using the packages: binom (Dorai‐Raj, 2014), car (Fox & Sanford, 2011), “ggplot2” (Wickham, 2016) and “survival” (Therneau, 2015). We did model selection using Wald Chi‐squared likelihood ratio tests, removing nonsignificant interactions. We retained all main effects and reported statistics of these from type II likelihood ratio tests using the “Anova” function from the “car” package (Fox & Sanford, 2011).

#### 2.4.1 Pupal survival after heat stress

We chose 36°C as our single experimental “hardening” temperature since it is the highest temperature that does not reduce fertility when males experience it for 4h (Parratt et al., 2021; Walsh et al., 2021). We analyzed pupal survival after heat stress using a logistic regression with survival as a Bernoulli response variable. Stress temperature, hardening treatment (nonhardened or hardened at 36°C), and their interaction were fitted as explanatory variables. To determine whether the hardening temperature altered its protective effect, we analyzed pupal survival of all flies hardened at 34, 35, and 36°C prior to heat stress at the key stress temperature of 40°C where protection is observed. We performed a logistic regression with survival as a Bernoulli response variable. We used hardening temperature as the explanatory variable. Note that the 34 and 35°C hardening temperatures were not measured at 37 and 38°C temperature stress at this preliminary stage, as these temperatures are nonlethal after a 4h stress (Walsh et al., 2021).

#### 2.4.2 Adult survival after heat stress

As every fly stressed at control temperatures (23°C) survived, we analyzed adult survival at the chosen stress temperature (38°C) only, using a logistic regression with survival as a Bernoulli response variable and sex (male or female), hardening treatment (nonhardened or hardened), and their interaction as explanatory variables.

#### 2.4.3 Pupal fertility over time

We analyzed the effect of heat stress on fertility over time with inverse Cox proportional hazard survival analyses (using the “survival” package (Therneau, 2015)). This allowed us to model the time in days posteclosion until focal individuals become fertile. We fit the first recorded time‐point at which fertility was observed (ARM) as our response variable with heat treatment (benign or stress), hardening treatment (nonhardened or hardened), and their interaction as independent variables.

#### 2.4.4 Adult fertility over time

We examined whether there was an immediate effect of heat stress on fertility, and whether hardening affects this response. We used a logistic regression with Day 1 fertility as a Bernoulli response variable and stress (benign or stressed), hardening treatment (nonhardened or hardened), and their interaction as explanatory variables.

Adult fertility over time was analyzed using two separate approaches due to the observed delayed sterility and how the experimental design was constructed around it. This allowed us to pull apart different hypotheses and test them. We first tested whether heat stress reduced fertility from Day 7 onward compared to benign temperature controls, due to delays in adult sterility. To do this, we used a mixed effect logistic regression on nonhardened flies, with fertility as a Bernoulli response variable and stress, time, and their interaction as explanatory variables. Fly ID was used as a random effect to account for nonindependence in the data.

We then tested whether hardening can improve fertility over time in stressed males. We used a mixed effect logistic regression on stressed flies, with fertility as a Bernoulli response variable and hardening, time, and their interaction as explanatory variables. Fly ID was used as a random effect to account for repeated measures in the data.

## 3 RESULTS

### 3.1 Survival after pupal heat stress

**FIGURE 1:**
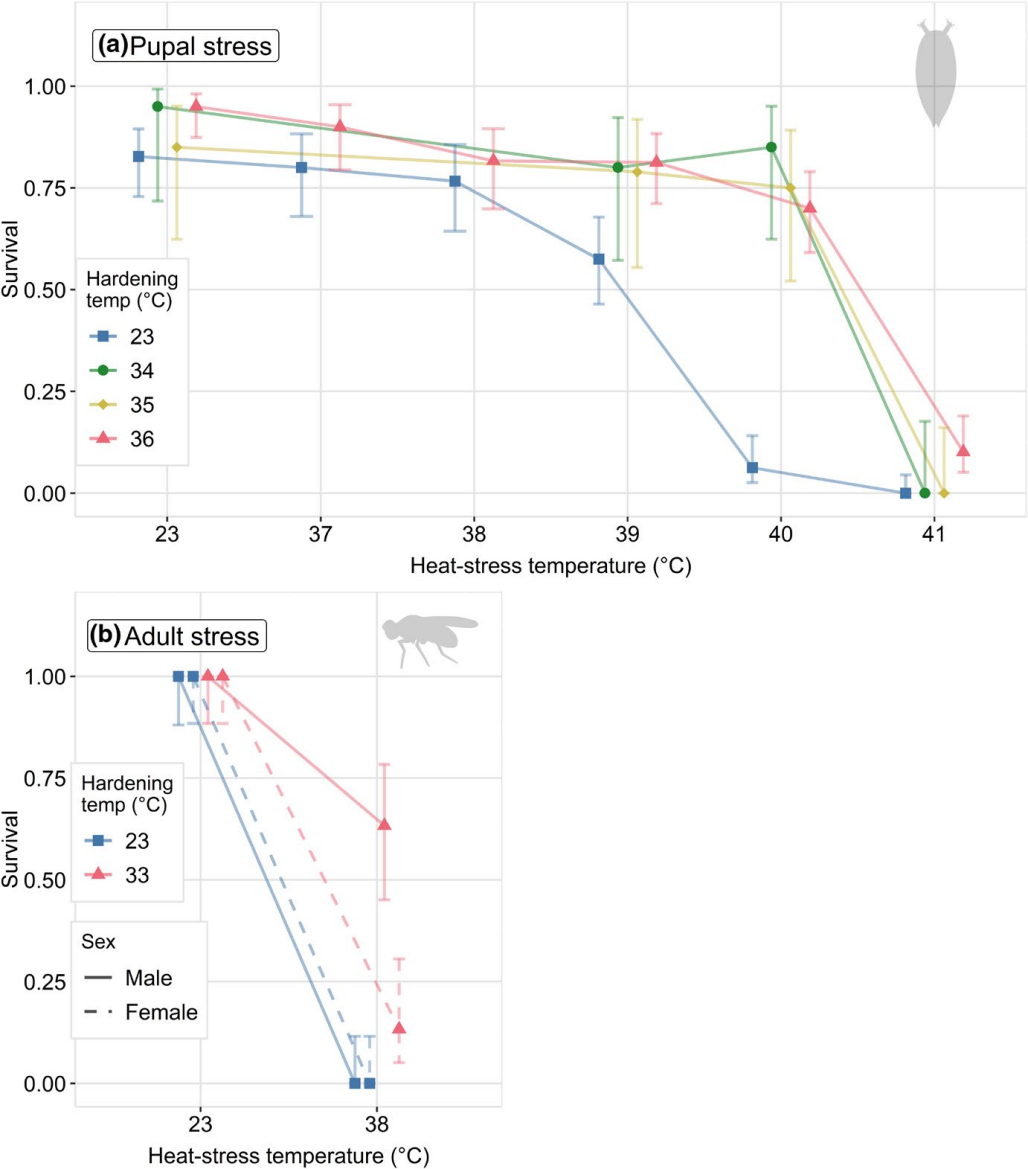
Proportion of surviving individuals after a 4‐h heat stress. Focal individuals were subjected to a prestress “hardening” treatment for 1 h immediately prior to temperature stress. (a) *D*. *virilis* individuals of unknown sex were heated during the pupal stage and subjected to a range of stressful temperatures. A range of hardening temperatures were also used to examine the hardening response. Note that the 34 and 35°C hardening temperatures were not measured at 37 and 38°C temperature stress. (b) Male and female *D*. *virilis* were heated during the adult stage 7 days postemergence, and subjected to two stress temperatures (23°C: benign, 38°C, stress). Error bars represent 95% confidence intervals

When focusing on a single hardening temperature (36°C) compared with nonhardened controls, we found that pupal survival probability was significantly affected by the interaction between hardening and heat stress temperature (χ^2^_(5)_ = 33.74, *p *< .001; Figure 1a). Specifically, pupae heat‐hardened at 36°C showed significantly improved survival at higher stress temperatures over nonhardened pupae. Between the 3 hardening temperatures of 34, 35, and 36°C, we found no effect of hardening temperature (χ^2^_(2)_ = 2.040, *p *= .361; Figure 1a) on individual survival at the pupal stress temperature of 40°C.

### 3.2 Survival after adult heat stress

There was no interaction between hardening and sex for adult survival at 38°C (χ^2^_(1)_ = 0.000, *p *= .999; Figure 1b). However, we found a main effect of hardening on survival (χ^2^_(1)_ = 41.321, *p *< .001; Figure 1b). Survival is significantly higher if adults have experienced a 1‐h hardening treatment at 33°C, as compared to nonhardened controls. We also found a main effect of sex, with lower survival in females than males (χ^2^_(1)_ = 16.891, *p *< .001; Figure 1b).

### 3.3 Fertility after pupal heat stress

**FIGURE 2:**
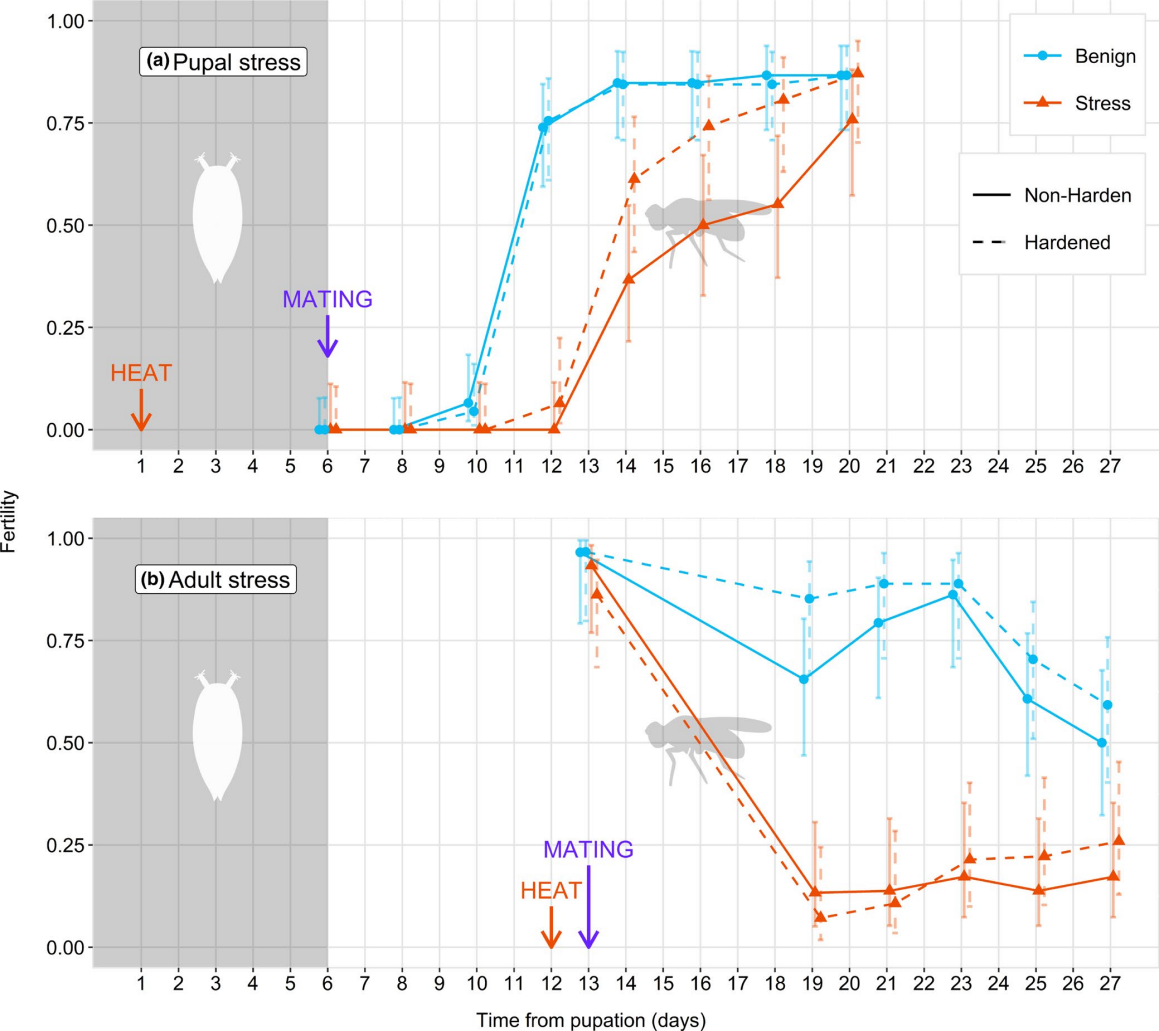
Cumulative fertility of male *D*. *virilis* over time after a 4‐h heat stress. Focal individuals were subjected to a prestress hardening treatment for 1h immediately prior to temperature stress. The age at heat stress is represented using an arrow, and the life stage of the individual is represented using gray (pupal) and white (adult) background. (a) Individuals were heated during the pupal stage at either benign (23°C) or stressful (38°C) temperatures. Individuals were exposed to a 1 h hardening treatment at 23°C (“nonhardening”) or 36°C (“hardening”) prior to heat stress. Focal males were given a single group of virgin females at the first day posteclosion. (b) Individuals were heated during the adult stage at either benign (23°C) or stressful (35°C) temperatures. Individuals were exposed to a 1 h hardening treatment at 23°C (“nonhardening”) or 33°C (“hardening”) prior to heat stress. Focal males were given access to 2 groups of virgin females: one from days 1 to 6 postheat, and another fresh set of virgin females from day 7 postheat, to account for delayed sterility of males. Error bars represent 95% confidence intervals

There was no interaction between pupal hardening and stress temperatures on the age of reproductive maturity (ARM) (Cox proportional hazard test interaction term: HR = 0.3831, χ^2^_(1)_ = 1.096, *p *= .295; Figure 2a). However, high pupal stress temperatures increase the time after eclosion until males can produce offspring (Cox proportional hazard test interaction term: HR = −0.8862, χ^2^_(1)_ = 23.27, *p *< .001; Figure 2a). This extends the ARM, with many males eventually becoming fertile. Pupal hardening does not significantly reduce ARM at the stress temperature of 38°C (Cox proportional hazard test interaction term: HR = 0.1034, χ^2^_(1)_ = 0.338, *p *= .561; Figure 2a).

### 3.4 Fertility after adult heat stress

Adult males were given an initial group of virgin females to mate with, and there was no interaction between stress temperature and hardening treatment on immediate fertility of adult males (χ^2^_(1)_ = 0.244, *p *= .621; Figure 2b, days 13–19). We also found no effect of heat stress on immediate fertility (χ^2^_(1)_ = 2.286, *p *= .130; Figure 2b, days 13–19), and no main effect of hardening on fertility at this initial time‐point (χ^2^_(1)_ = 0.590, *p *= .443; Figure 2b, days 13–19).

From 7 days postheat stress onward in nonhardened flies, there was no interaction between heat stress and time (χ^2^_(1)_ 3.333, *p *= .068; Figure 2b, days 19–27). However, we found that heat stress significantly reduced fertility through a main effect of stress (χ^2^_(1)_ = 28.444, *p *< .001; Figure 2b, days 19–27). Stressed males had lower fertility than controls after 7 days postheat stress. We found no significant effect of time on fertility after day 7 (χ^2^_(1)_ = 2.413, *p *= .120; Figure 2b, days 19–27) meaning fertility remained low post 7 days.

There was no interaction between hardening and time on fertility at the stress temperature of 34°C when measured after day 7 (χ^2^_(1)_ = 2.1824, *p *= .140; Figure 2b, days 19–27). Hardening also did not affect fertility of heat‐stressed adults (χ^2^_(1)_ =0.1319, *p *= .717; Figure 2b, days 19–27) meaning hardening does not change the sterility pattern induced by thermal stress, even though there was a main effect of time on fertility (χ^2^_(1)_ = 4.265, *p *= .039; Figure 2b, days 19–27), where fertility increased slightly as the experiment progressed.

## 4 DISCUSSION

We found functionally different impacts of thermal stress at different life‐history stages on fertility in *Drosophila virilis*. Pupal heat stress delays the age of reproductive maturity (ARM), whereas adult heat stress sterilizes most males. Many stressed adult males are fertile immediately postheat stress but lose fertility over a week and remain permanently sterile for the duration measured. Heat‐induced sterility in *Drosophila melanogaster* has been associated with disruptions to spermatid elongation during spermatogenesis (Rohmer et al., 2004). Therefore, it is possible that mature sperm stored in the seminal vesicles of adult males are relatively unharmed and can be used by stressed males, whereas immature sperm are destroyed and the capacity to produce sperm is disrupted. However, it is unclear why pupae appear to recover fertility over the course of the experiment, whereas adults remain sterile. Benign adult males saw a drop‐off in fertility over the last two time‐points. Therefore, it is possible that the combination of heat‐induced sterility and natural aging prevent heated adult males from recovering fertility over the experiment. Exploring how fertility is affected by high temperature at the pupal and adult stages by looking at sperm production over an individual's lifetime may be necessary to disentangle these differences.

We found pupae were more thermally robust than adults. At 38°C, nonhardened adult *D*. *virilis* cannot survive, whereas pupae show high survival, and their ARM is delayed but eventually recovers. Pupae are immobile, so high physiological thermal tolerance may be particularly important for pupae as they cannot behaviorally thermoregulate to escape heat stress. However, the finding that pupae are more resistant to thermal stress than other life stages contrasts with some previous studies. For example, a recent study examining flour beetles found that pupae and immature males are the most vulnerable life stages to both fertility loss and survival at high temperatures (Sales et al., 2021). Additionally, nonhardened *D*. *melanogaster* pupae have very similar upper lethal limits than adults (Moghadam et al., 2019). Similarly in yellow dung flies (*Scathophaga stercoraria*), there is no simple relationship between heat tolerance and mobility of life stage, with early and late‐stage pupae showing contrasting responses to thermal stress (Blanckenhorn et al., 2014). With no obvious pattern in how life stage interacts with heat‐induced death and sterility across species groups, it is clear that studies on thermal limits should consider examining all life stages that are likely to be exposed to high temperatures in the wild.

As expected, we found *D*. *virilis* can improve high temperature survival through prior hardening at sublethal stress temperatures. This response occurs in both life history stages measured. The effect is sex‐specific in adults such that heat‐hardened males show higher survival over heat‐hardened females at lethal temperatures. A meta‐analysis on sex differences in acclimation capacity, including four *Drosophila* species, found no significant differences in overall acclimation capacity between males and females (Pottier et al., 2021). However, the authors found that where differences between sexes exist, females appear to have higher acclimation capacity than males. It has previously been shown that *D*. *virilis* female fertility is robust to high pupal temperatures when compared with male fertility (Walsh et al., 2021). It follows that females would be able to utilize the improved survival at high temperatures by reproducing. This makes the finding that heat‐hardened males actually show higher survival than females surprising, as it is difficult to see the fitness benefit gained by permanently sterilized males surviving high temperatures.

In contrast to survival, we found no significant protective impact of this same hardening treatment on fertility at sterilizing temperatures. This is true for both pupae and adults, suggesting that, although prior heat‐hardening improves survival at lethal temperatures, it does not protect male fertility. Whereas previous studies found a positive impact of heat‐hardening on reproduction (Jørgensen et al., 2006), here we find no measurable benefit of heat‐hardening on fertility. While we demonstrated that a range of heat‐hardening temperatures can protect survival, we chose a single heat‐hardening treatment when testing whether heat‐hardening also protects pupal and adult fertility. So we do not claim that there is no heat‐hardening treatment that might protect fertility in this species. Rather, our point is that a hardening temperature that gives clear survival benefits does not appear to provide any defense for fertility. This suggests that lessons about how hardening protects survival under thermal stress cannot be directly applied to fertility.

We tested relatively short periods of hardening and stress, but longer‐term acclimation to high temperatures can influence reproduction. In the flour beetle *Tribolium castaneum*, adult male development at stressful temperatures results in males producing sperm with shorter tails (Vasudeva et al., 2019). This is shown to be an adaptive morphological shift, with shorter sperm doubling performance when males are reproducing at high temperatures. Similarly, a recent study in *D*. *melanogaster* found that a three‐day acclimation period prior to mating increases mating success by around 70% at stressful temperatures (Stazione et al., 2019). It is known that the timing of heat shock and heat‐hardening/acclimation can drive differences in the response to temperature stress (Weldon et al., 2011; Zhang et al., 2021). Possibly, there is a delay for any physiological response to “kick‐in” before components of fertility can be protected. Many experiments demonstrating thermal plasticity of reproductive traits utilize multiple‐day stress treatments (Stazione et al., 2019; Vasudeva et al., 2019), or delays between “hardening” and thermal stress (Jørgensen et al., 2006). We did not provide our flies with such a gap, immediately moving them from hardening to stress temperatures, which might have impaired any hardening effect. Indeed, natural populations may experience more gradual transitions across sublethal and lethal temperatures. These may result in recovery periods between heat‐hardening and stressful temperatures, or allow organisms to more gradually transition between temperatures. However, it is also possible that natural populations caught during the peak midday sun of a heatwave may not realistically have the opportunity to “ramp‐up” their physiological response. Clearly plasticity in reproductive traits is possible; however, its general capacity to allow organisms to cope with climate change is still unclear (Sgrò et al., 2016). If a similar lack of strong or robust short‐term heat‐hardening for fertility is found across taxa, then organisms may be more vulnerable to climate change than previously thought.

There are a few notable caveats to our findings that should be taken into consideration when evaluating how species will respond to extreme temperature stress through plasticity. A more detailed experiment in which males were provided with virgin females at shorter intervals may show some weak effects of hardening for fertility that we did not pick up with our design. In addition, our work has focused almost exclusively on high temperature stress. While this is clearly important in a warming world, climate models also suggest cold stress will also increase for many organisms, as snow cover is reduced, and winters become harsher in some areas. Studying how cold stress impacts on fertility and sterility is both urgently needed, and fortunately more developed than sublethal impacts of high temperature stress.

Superficially, it seems that improving survival of males via heat‐hardening may be less beneficial to fitness than previously thought, given that males may be alive but permanently sterilized. Parratt et al. (2021) found that males from 19 of 43 Drosophila species could survive apparently permanently sterilizing temperatures, suggesting there must be a biological explanation. The adaptive benefit of heat‐hardening is particularly confusing if it protects survival without allowing individuals any opportunities to reproduce. However, a key finding here is that both life stages measured still have a limited capacity to reproduce after heat shock. Males heated as pupae are eventually sexually mature and heated adult males can reproduce within a few days, before long‐term sterility manifests. Therefore, the improved survival at extreme temperatures may provide more males with these limited opportunities to use up surviving mature sperm, without protecting reproductive traits directly. It is also possible that if males sterilized as adults were kept long term, they may restore some fertility over time. Alternatively, male hardening could simply be a neutral by‐product of selection on females for survival at high temperatures, as females are far better able to maintain fertility at near‐lethal temperatures (Walsh et al., 2021).

To gain a more complete understanding of how natural populations will be affected by heat‐waves, measuring the difference of survival and fertility between life stages will be important. Our findings also suggest that research needs to consider that heat‐hardening may not be a sufficient plastic rescue mechanism, although heat‐hardening effects on fertility in more taxa need to be tested. Importantly, studies showing the positive effects of heat‐hardening should consider whether surviving individuals are fully fertile. This will allow researchers to more fully understand the adaptive benefits of these responses.

## CONFLICT OF INTEREST

None declared.

## AUTHOR CONTRIBUTIONS

**Benjamin S. Walsh:** Conceptualization (lead); Data curation (lead); Formal analysis (lead); Investigation (lead); Methodology (lead); Visualization (lead); Writing – original draft (lead); Writing – review & editing (lead). **Steven R. Parratt:** Conceptualization (supporting); Formal analysis (supporting); Methodology (supporting); Visualization (supporting); Writing – review & editing (supporting). **Natasha L. M. Mannion:** Methodology (supporting); Writing – review & editing (supporting). **Rhonda R. Snook:** Writing – review & editing (supporting). **Amanda Bretman:** Writing – review & editing (supporting). **Tom A. R. Price:** Conceptualization (equal); Funding acquisition (lead); Methodology (supporting); Project administration (lead); Resources (lead); Writing – original draft (equal); Writing – review & editing (equal).

### OPEN RESEARCH BADGES

This article has earned an Open Data Badge for making publicly available the digitally‐shareable data necessary to reproduce the reported results. The data is available at https://doi.org/10.5061/dryad.xd2547dhg.

#### 1 DATA AVAILABILITY STATEMENT

The data and analysis code are available at: https://doi.org/10.5061/dryad.xd2547dhg
